# Leveraging machine learning tools and algorithms for analysis of fruit fly morphometrics

**DOI:** 10.1038/s41598-022-11258-w

**Published:** 2022-05-03

**Authors:** Daisy Salifu, Eric Ali Ibrahim, Henri E. Z. Tonnang

**Affiliations:** 1 International Centre of Insect Physiology and Ecology (icipe), P.O. Box 30772-00100, Nairobi, Kenya; 2 Department of Statistics, Jomo Kenyatta University of Agriculture and Technology, P.O. Box 62000-00200, Nairobi, Kenya

**Keywords:** Statistics, Machine learning

## Abstract

Analysis of landmark-based morphometric measurements taken on body parts of insects have been a useful taxonomic approach alongside DNA barcoding in insect identification. Statistical analysis of morphometrics have largely been dominated by traditional methods and approaches such as principal component analysis (PCA), canonical variate analysis (CVA) and discriminant analysis (DA). However, advancement in computing power creates a paradigm shift to apply modern tools such as machine learning. Herein, we assess the predictive performance of four machine learning classifiers; K-nearest neighbor (KNN), random forest (RF), support vector machine (the linear, polynomial and radial kernel SVMs) and artificial neural network (ANNs) on fruit fly morphometrics that were previously analysed using PCA and CVA. KNN and RF performed poorly with overall model accuracy lower than “no-information rate” (NIR) (*p* value > 0.1). The SVM models had a predictive accuracy of > 95%, significantly higher than NIR (*p* < 0.001), Kappa > 0.78 and area under curve (AUC) of the receiver operating characteristics was > 0.91; while ANN model had a predictive accuracy of 96%, significantly higher than NIR, Kappa of 0.83 and AUC was 0.98. Wing veins 2, 3, 8, 10, 14 and tibia length were of higher importance than other variables based on both SVM and ANN models. We conclude that SVM and ANN models could be used to discriminate fruit fly species based on wing vein and tibia length measurements or any other morphologically similar pest taxa. These algorithms could be used as candidates for developing an integrated and smart application software for insect discrimination and identification. Variable importance analysis results in this study would be useful for future studies for deciding what must be measured.

## Introduction

Analysis of landmark-based morphometric measurements taken on body parts of insects have been a taxonomic approach alongside DNA barcoding in detecting morphological differences to discriminate closely related species, justify synonyms, demonstrate morphological variation across landscapes, altitudinal or geo-graphical gradients and propose new species^1–3^. The measurements are usually of multivariate nature requiring multi-variate analysis techniques to be able to classify each specimen to a specific group. Analysis of morphometric measurements have been deemed as a viable alternative to the complicated and time-consuming taxonomic skills required in insect identification. Many studies have measured wing characteristics such as wing venation^2,4^ and wing geometry^5–7^ as landmark for identification of insects. Morphometrics data have in many cases produced results congruent with phylogenetic groupings from DNA sequencing and hence morphometrics have gained popularity.

Conventional classification analysis approaches have been used to analyse morphometric measurements namely principal components analysis (PCA), discriminant analysis (DA), canonical variate analysis (CVA), cluster analysis (CA) just to mention a few. Hernández-Ortiz et al.^8^ used DA and CA on morphometrics variables of the acuelans¸ wing and menosotum to distinguish populations of *Anastrepha fraterculus* complex. Billah et al.^9^ analyzed morphometric measurements of allopatric populations of fruit fly parasitoids from coffee fields using PCA and CVA where results showed that the relationship between the populations was corroborated by genetic evidence from amplified fragment length polymorphism (AFLP) data. A study by Khamis et al.^2^ used PCA and CVA to distinguish *Bactrocera* species collected from various countries to establish whether *B. invadens* samples collected from Africa could be distinguished from Asian *Bactrocera* species based on wing vein and tibia length morphometrics alongside DNA barcoding. The study showed some level of concordance between molecular and morphometric results. While conventional machine learning methods such as k-means cluster analysis^10^, PCA^2,3^, discriminant analysis^6^, canonical variate analysis^5^, have been widely used, modern machine learning techniques are gradually gaining popularity for morphometrics in insect science. For instance, the k-nearest neighbors^11^, artificial neural network^12^ and random forest^13^ algorithms were recently used for morphometrics of insects. While conventional methods are largely parametric in nature allowing distributional assumptions, modern machine learning techniques are mainly non-parametric, thus they do not make assumptions about the kind of mapping functions between output and input variables. Consequently, the novel algorithms are more robust in their performance. The objective of the present study is therefore to assess the predictive performance of four modern machine learning classifiers; K-nearest neighbor (KNN), random forest (RF), support vector machine (SVM) and artificial neural network (ANN) on morphometric measurements on fruit fly, *Bactrocera* spp and determine the variable importance (VI) of predictor variables. Such information would be useful for the development of an integrated and smart application software for insect discrimination and identification.

## Results

### The k-nearest neighbor classifier

The optimal value for the tuning parameter k for kNN classification model was selected based on highest model accuracy on training data for a range of k values. Model accuracy reduced with increasing k values. Accuracy was highest for k = 5 (Table 1).

**Table 1:** Values of the tuning parameter, k and the corresponding accuracy and kappa statistics for the kNN model on the training dataset.

k	Accuracy	Kappa
5	0.927	0.639
7	0.924	0.615
9	0.915	0.564
11	0.908	0.510
13	0.904	0.483
15	0.897	0.430
17	0.893	0.399
19	0.889	0.367
21	0.887	0.348
23	0.884	0.324

The kNN classifier model with k = 5 had a predictive accuracy rate of 0.932 [95% CI: 0.889, 0.957] and “no-information rate” (NIR) of 0.929 with* p* value (accuracy > NIR) = 0.991, thus there is no evidence accuracy is higher than NIR, suggesting that the predictive performance of the kNN classifier on the data is not any better than random guessing. We cannot use this model to predict for new data.

### The RF classifier

**Figure 1:**
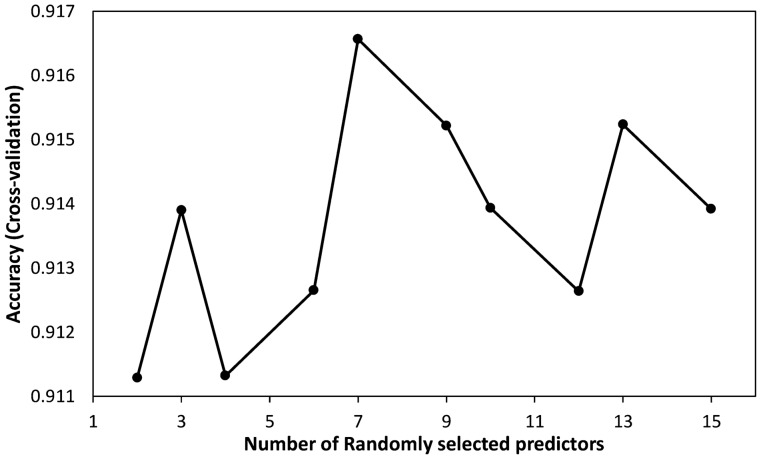
Variation in accuracy for number of randomly selected predictor variables (*mtry*) for the random forest classifier. Model accuracy is highest for *mtry* = 7.

The RF hyperparameter, *mtry* was evaluated for the RF model using repeated cross-validation and *mtry* equal to 7 was optimal. This means that the RF classifier used 7 predictors to split the tree. Graphical presentation of the results on accuracy against randomly selected predictors is as shown in Fig. 1.

The RF classifier model had an overall accuracy of 0.911 [95% CI: 0.874, 0.939], kappa statistic of 0.54 and NIR of 0.929 with p-value (accuracy > NIR) = 0.916 suggesting a poor model. We therefore do not pursue the confusion matrix.

### Support vector machine (SVM) classifier

Three SVM classifier models were implemented; linear kernel SVM, polynomial kernel SVM and radial basis function SVM and here we provide the predictive performance of these models respectively.

#### Linear kernel SVM

**Figure 2:**
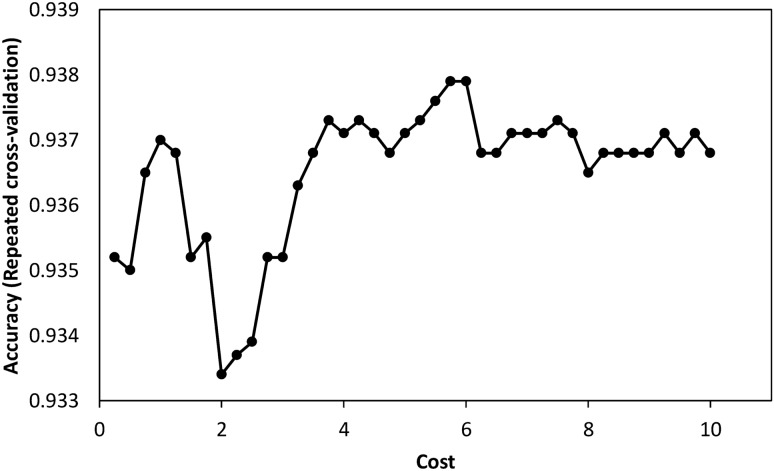
Linear SVM model accuracy (y-axis) for values of cost parameter (x-axis) obtained from the repeated cross-validation of the training sample data. Cost “C” = 5.75 gives the optimal model.

The linear SVM model attained highest accuracy with cost “C” of 5.75. This cost parameter was obtained using repeated cross-validation whose results are shown in Fig. 2.

The linear kernel SVM classification model (C = 5.75) had overall accuracy of 0.957 [95% CI: 0.929, 0.976]. The corresponding NIR was 0.886 with p-value < 0.0001 (accuracy > NIR), thus accuracy score was significantly higher than NIR which implies that the classifier model performed better than one could do by always predicting the most common class. The model had a Kappa of 0.811 signifying substantial strength of agreement between the model’s predictions and the actual labels of classes while controlling for accuracy of a random classifier. The AUC of the receiver operating characteristics for the linear kernel SVM classifier was 0.911. Table 2 displays the classifier predictions on the test dataset and classifier metrics based on the confusion matrix. From the predictions, it is clear all samples of *B. oleae* (Bol), and *B. zonata* (Bzo) in the test dataset have been classified into their respective observed group. The linear kernel SVM model achieved sensitivity rate of above 80% for all species except for *B. dorsalis* (Bdo) while specificity ranged from 89 to 100%.

**Table 2:** Classification results for the SVM classifiers on test dataset of morphometric measurements of *Bactrocera spp*., with observed species affiliation in the rows and predicted species allocation in the columns. Correct classification rate appears along the diagonal in bold.

Classifier	Observed	Predicted (%)							Sensitivity	Specificity
		Bco	Bcu	Bdo	BI	Bka	Bol	Bzo		
SVM-L	Bco	**80.0**	0	0	20.0	0	0	0	1.000	0.997
	Bcu	0	**100**	0	0	0	0	0	0.818	1.000
	Bdo	0	0	**25.0**	75.0	0	0	0	0.500	0.981
	BI	0	0.7	0.7	**98.6**	0	0	0	0.965	0.892
	Bka	0	0	0	37.5	**62.5**	0	0	1.000	0.991
	Bol	0	0	0	0	0	**100**	0	1.000	1.000
	Bzo	0	0	0	0	0	0	**100**	1.000	1.000
SVM-R	Bco	**80.0**	0	0	20.0	0	0	0	1.000	0.997
	Bcu	0	**88.9**	0	11.1	0	0	0	1.000	0.997
	Bdo	0	0	**37.5**	62.5	0	0	0	1.000	0.984
	BI	0	0	0	**100**	0	0	0	0.956	1.000
	Bka	0	0	0	75.0	**25.0**	0	0	1.000	0.981
	Bol	0	0	0	0	0	**100**	0	1.000	1.000
	Bzo	0	0	0	0	0	0	**100**	1.000	1.000
SVM-P	Bco	**80.0**	0	0	20.0	0	0	0	0.800	0.997
	Bcu	0	**88.9**	0	11.1	0	0	0	0.889	0.997
	Bdo	0	0	**50.0**	50.0	0	0	0	1.000	0.988
	BI	0.35	0.35	0	**98.2**	1.1	0	0	0.962	0.865
	Bka	0	0	0	62.5	**37.5**	0	0	0.500	0.984
	Bol	0	0	0	0	0	**100**	0	1.000	1.000
	Bzo	0	0	0	0	0	0	**100**	1.000	1.000

Bco—*B. Correcta*, Bcu—*B****.**** cucurbitae*, Bdo—*B. dorsalis*, BI—*B. invadens*, Bka—*B. kandiensis*, Bol—*B. oleae*, Bzo—*B. zonata;* SVM-L: linear kernel SVM, SVM-R: radial kernel SVM, SVM-P: polynomial kernel SVM.

#### Radial kernel SVM classifier

Selection of optimal model for radial kernel SVM requires determination of the optimal values of tuning parameters namely gamma (**γ**) and cost (C). We tested different values of **γ** ranging from 0.01 to 0.1 with step 0.01 while C was in range 0.01 to 10.0 with step 0.25 and obtained the values that minimize the classification error for the tenfold cross-validation. The optimal model was obtained with **γ** = 0.06 and C = 9.51. Using these parameters, the radial kernel SVM model had accuracy of 0.96 [95% CI: 0.933, 0.978], Kappa statistic of 0.810 and NIR of 0.91 with p-value (accuracy > NIR) = 0.0002. NIR being significantly lower than accuracy, suggests the radial kernel SVM model is superior to random guessing. The AUC of the receiver operating characteristics for the radial kernel SVM classifier was 0.933.

Just as with the linear kernel SVM model, the sensitivity and specificity for *B. oleae* (Bol), and *B. zonata* (Bzo) was 100%. (Table 2).

#### Polynomial SVM classifier model

The polynomial SVM model attained optimal accuracy at a degree of 2, scale of 2 and cost of 0.1. Using the test dataset, the classifier model yielded predictive accuracy of 0.951 [95% CI: 0.921, 0.972], Kappa statistic of 0.784 and NIR of 0.886 with p-value (accuracy > NIR) < 0.0001, suggesting a good model. The AUC of the receiver operating characteristics for the polynomial kernel SVM classifier was 0.959. The sensitivity for *B. oleae* (Bol), *B. zonata* (Bzo) and *B. dorsalis* (Bdo) was 100% respectively, while the model had smallest sensitivity on *B. kandiensis* (Bka) (Table 2).

### Artificial neural network (ANN) classifier

The optimal ANN model was selected based on the accuracy obtained by varying the number of nodes of the network. The ANN model was optimal at 17 nodes and decay of 0.042. We fitted a feedforward (15-17-7) network, thus a model with 15 input neurons, 17 hidden neurons and 7 input neurons. The predictive accuracy for this model was 0.96 [95% CI: 0.933, 0.979], Kappa statistic of 0.833 and NIR of 0.873 with p-value (accuracy > NIR) < 0.0001. Thus, the neural network was superior to NIR. The AUC of the receiver operating characteristics for the ANN model was 0.986. The classification results of the ANN classifier on test dataset and the estimated metrics are presented in Table 3. From the predictions, it is clear that all samples of *B. Correcta* (Bco), *B. oleae* (Bol), and *B. zonata* (Bzo) in the test dataset have been classified into their respective observed group. The metrics for ANN classifier suggests that sensitivity was lowest for *B. dorsalis* (Bdo) and *B. kandiensis* (Bka) while the sensitivity and specificity for *B. Correcta* (Bco), *B*. *oleae* (Bol) and *B*. *zonata* (Bzo) was 100%, respectively (Table 3).

**Table 3:** Classification results for the ANN classifier on test dataset of morphometric measurements of *Bactrocera* spp., with observed species affiliation in the rows and predicted species allocation in the columns. Correct classification rate appears along the diagonal in bold.

Observed	Predicted (%)							Sensitivity	Specificity
	Bco	Bcu	Bdo	BI	Bka	Bol	Bzo		
Bco	**100**	0	0	0	0	0	0	1.000	1.000
Bcu	0	**88.9**	0	11.1	0	0	0	0.889	0.997
Bdo	0	0	**50.0**	50.0	0	0	0	0.667	0.987
BI	0	0.35	0.35	**98.2**	1.1	0	0	0.975	0.878
Bka	0	0	12.5	25.0	**62.5**	0	0	0.625	0.991
Bol	0	0	0	0	0	**100**	0	1.000	1.000
Bzo	0	0	0	0	0	0	**100**	1.000	1.000

Bco—*B. Correcta*, Bcu—*B****.**** cucurbitae*, Bdo—*B. dorsalis*, BI—*B. invadens*, Bka—*B. kandiensis*, Bol—*B. oleae*, Bzo—*B. zonata.*

Finally, a summary of performance metrics namely accuracy, Kappa, no-information rate (and associated p-values), and AUC of the ML classifiers under study are presented in Table 4. AUC is only estimated for the best classifiers, SVM and ANN.

**Table 4:** Summary of performance metrics for all the machine learning classifiers under study.

Classifier model	Accuracy [95% CI]	Kappa	NIR	*p* value	AUC
				(Acc > NIR)	
k-Nearest Neighbor	0.932 [0.899, 0.957]	0.648	0.929	0.469	
Random Forest	0.912 [0.874, 0.939]	0.536	0.929	0.916	
**SVM**					
Linear kernel	0.957 [0.929, 0.976]	0.811	0.886	< 0.0001	0.911
Radial kernel	0.960 [0.933, 0.979]	0.810	0.908	0.0002	0.933
Polynomial kernel	0.951 [0.921, 0.972]	0.784	0.886	< 0.0001	0.959
ANN	0.960 [0.933, 0.979]	0.827	0.883	< 0.0001	0.986

*NIR* no-information rate,* Acc* accuracy,* AUC* Area under the curve of the receiver operating characteristics,* SVM* Support vector machine,* ANN* Artificial neural network.

### Variable importance for the predictor morphometric measurements

**Figure 3:**
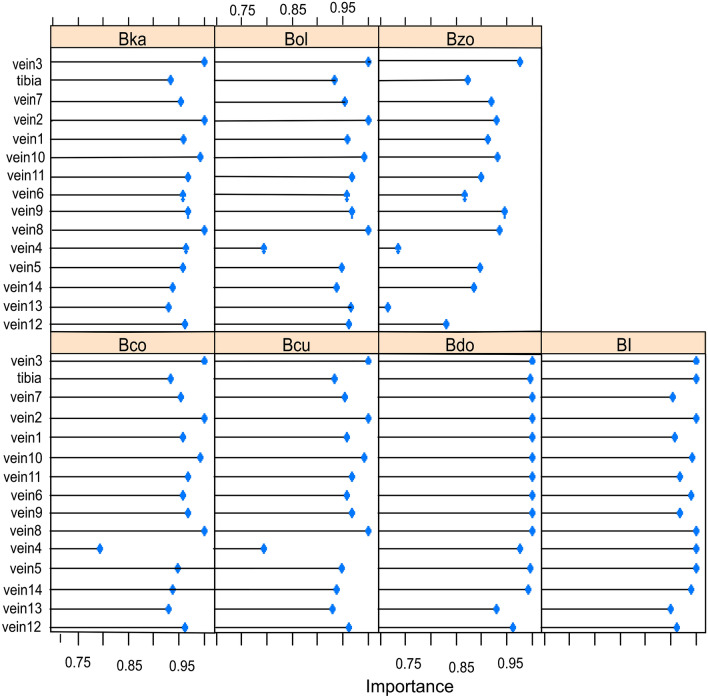
Analysis of variable importance (VI) for the radial kernel SVM model. Veins 3, 2, 8, and 10 are identified as predictors of higher importance than others in all species except for Bdo (*B. dorsalis*) and BI (*B. invadens*).

The relative importance of variables was obtained for the SVM models and ANN model. All SVM models; linear kernel, radial kernel and polynomial kernel SVM gave similar results in terms of variable importance. The SVM models identified veins 3, 2, 8 and 10 (in that order) as of higher importance than others in all species except for *B. invadens* (BI) and *B. dorsalis* (Bdo) where the models suggest that almost all predictor variables are of high importance, including veins 2, 3, 8 and 10 (Fig. 3). The VI graphical displays for the linear kernel and polynomial kernel SVMs are given as supplementary material (Supplementary Fig. S1 and Supplementary Fig. S2).

**Figure 4:**
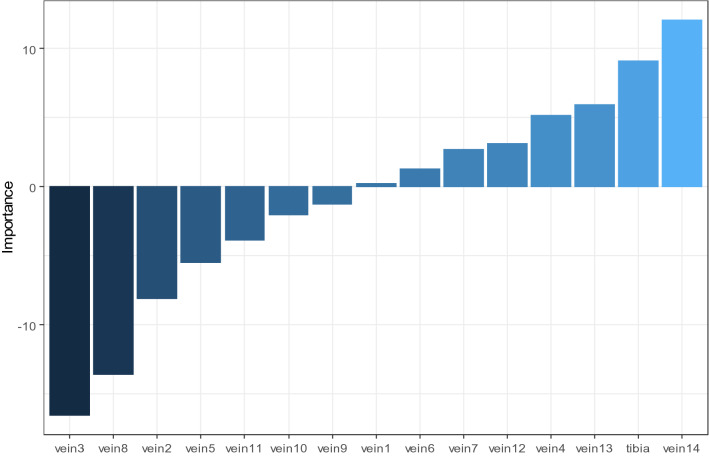
Analysis of variable importance (VI) for the artificial neural network model. Veins 3, 8, 2, 14 and tibia length are analysed as of higher importance than others in predicting the *Bactrocera* spp.

The variable importance (VI) results for the ANN model are displayed in Fig. 4. ANN model introduced vein 3, 8, 2, 14 and tibia length as predictors of higher importance than others.

## Discussion

This paper evaluates novel analysis techniques, herein machine learning tools and algorithms namely, KNN, RF, SVM and ANN to classify fruit fly species based on morphometrics data that were previously analysed using conventional statistical methods. Although conventional classification methods are very popular in agricultural sciences^5,10^, advancement in data science and computing power provide an opportunity to harness and integrate the novel and robust machine learning tools as analytics routine in insect science research as demonstrated by this example on morphometrics.

KNN and RF classifiers performed poorly with ‘no-information rate’ being higher than overall accuracy with p-value > 0.05, thus the models were no better than random guessing in the classification of *Bactrocera* spp. Millard and Richardson^14^ showed that random forest models improve with larger training datasets. The RF classifier must have suffered even more from the small training samples of the minority classes leading to poor predictive performance. SVM and ANN models were superior to KNN and RF in that all the SVM models, namely linear kernel SVM, Polynomial kernel SVM and Radial kernel SVM, had overall accuracy of above 95% and AUC of > 91 and ANN had overall accuracy of 96% and AUC of 0.986 with ‘no-information rate’ significantly lower than accuracy for both ANN and the SVM models. The superiority of SVM in terms of accuracy was also shown in a study by Smoliński et al.^15^ in which two traditional machine learning classifiers (linear and quadratic discriminant classifiers) and four modern machine learning classifiers; kNN, Classification and regression trees, RF and SVM were used to discriminate stocks of fish species based on otolith shape.

Among the three forms of the SVM models, the linear kernel SVM (accuracy 95.7%, AUC = 0.911) and radial kernel SVM model (accuracy 96.0%, AUC = 0.933) had kappa values higher than the polynomial kernel (accuracy 95.1%, AUC = 0.959). This study makes a very narrow distinction on predictive performance among the three SVM models while Nguyen^16^ who compared linear, polynomial and radial kernel SVM regression models concluded that the radial basis function was more appropriate than linear and polynomial kernel functions in predicting blast-induced ground vibration in an open-pit coal mine.

The data used in this study were initially analysed using principal component analysis (PCA) and canonical variate analysis (CVA) alongside DNA barcoding in Khamis et al.^2^. We therefore compare the classification of our best models with that obtained by DNA barcoding. Our best-chosen models, SVM and ANN predicted *B. oleae* and *B. zonata* as distinct groups while misclassification was largely among the three species *B. kandiensis*, *B. invadens* and *B. dorsalis.* These findings concur with results of DNA barcoding in Khamis et al. ^2^ and supported by mahalanobis squared distance which was smallest between *B. invadens* and *B. dorsalis* (11.4) and *B. invadens* and *B. kandiensis* (8.1) as compared to distance between *B. invadens* and *B. zonata* (43.1) and *B. invadens* and *B. oleae* (45.1).

PCA is a linear transformation of data from multiple axis to principal component axis. The principal components as new axis provides the best angle to see and evaluate the data such that any hidden group structures are revealed. On the other hand, Canonical variate analysis is similar to PCA but assumes that the group structure in the observations is known a *priori*. As applied on the morphometrics data, PCA and CVA were purely descriptive and graphical while the machine learning techniques being assessed in this paper provide model’s performance measures such as accuracy, kappa, area under curve, no-information rate and hence superior to the conventional methods used previously. This superiority is well pronounced when the data available are balanced, and it is usually recommended to select an algorithm based on the available datasets. In other words, the poor predictions observed with KNN and RF are not directly resulting from the predictive ability of the algorithms, but it is rather a result of the type and quantity of dataset. Techniques such as RF is non-linear and known to perform extremely well with large and noisy datasets. Often, it is advisable to first apply PCA to clean the data prior to running this algorithm. PCA has the advantage that it is easy to implement and is purely descriptive.

This paper has further provided information on variable importance that was not previously provided for these data, thus our best machine learning classifiers, SVM and ANN have analysed wing veins 3, 2, 8, 10, 14 and tibia length as predictor variables of higher importance than others. This information could be useful for future studies.

SVM and ANN algorithms achieved the highest predictive accuracy for the fruit fly morphometric measurements with NIR lower than accuracy and thus our choice of classifiers for these data. However, we recommend that discrimination studies should test a range of machine learning classifiers because the selection of the best-performing algorithms can be case-specific and depends, for instance, on the number of classes, similarity between groups, or type and number of variables in the dataset^17^. We subjected our ML models to multi-class imbalanced data. In as much as SVM and ANN produced good results, we recommend the use of data generation mechanisms to generate synthetic samples to boost samples for the minority classes.

The findings of our study suggest that SVM and ANN algorithms are a good alternative to conventional statistical classifiers and can be used to discriminate fruit fly species based on wing vein measurements and tibia length or any other morphologically similar pest taxa. These algorithms could be used as candidates for developing an integrated and smart application software for insect discrimination and identification. The VI results in this study would be useful for future studies for deciding what must be measured.

## Materials and methods

### Description of the data

This study used secondary data on measurements of wing veins and tibia length of male samples of fruit fly *Bactrocera* spp collected from various parts of Africa and Asia. Specimen were collected for *Bactrocera invadens*, *Bactrocera correcta*, *Bactrocera cucurbitae*, *Bactrocera dorsalis*, *Bactrocera kandiensis*, *Bactrocera oleae*, and *Bactrocera zonata*. Fourteen wing vein distances between 15 selected landmarks of the right wing and right hind tibia length were measured. A full description of the data is found in Khamis et al.^2^. The summarized data on 14 wing vein measurements and tibia length (mm) are in Table 5.

**Table 5:** Mean measurements of wing vein distances and tibia length (mm) of fruit fly (*Bactrocera *spp.) specimen collected from African countries and Asia.

Variable	*Bactrocera *spp.						
	Bco	Bcu	Bdo	BI	Bka	Bol	Bzo
Vein 1	4.086	5.115	4.211	4.748	4.947	3.585	4.334
Vein 2	0.631	0.871	0.719	0.746	0.749	0.612	0.641
Vein 3	1.022	1.382	1.175	1.284	1.343	0.876	1.195
Vein 4	0.503	0.548	0.517	0.545	0.605	0.316	0.616
Vein 5	1.265	1.584	1.351	1.497	1.591	1.018	1.510
Vein 6	0.384	0.504	0.412	0.444	0.488	0.291	0.399
Vein 7	1.761	2.150	1.891	2.067	2.156	1.549	1.943
Vein 8	0.621	0.865	0.706	0.772	0.789	0.544	0.679
Vein 9	0.701	0.913	0.770	0.878	0.907	0.653	0.727
Vein 10	0.962	1.332	1.094	1.191	1.263	0.844	0.981
Vein 11	2.160	2.726	2.291	2.489	2.641	1.940	2.306
Vein 12	1.120	1.356	1.151	1.229	1.270	1.114	1.116
Vein 13	1.078	1.340	1.051	1.150	1.251	0.938	1.186
Vein 14	2.054	2.500	2.156	2.362	2.409	1.689	2.165
Tibia length	1.471	1.728	1.522	1.679	1.721	1.153	1.506

Bco—*B. Correcta* (n = 18), Bcu—*B. cucurbitae* (n = 31), Bdo—*B. dorsalis* (n = 28), BI—*B. invadens* (n = 940), Bka—*B. kandiensis* (n = 28), Bol—*B. oleae* (n = 28), Bzo—*B. zonata* (n = 18).

### Machine learning algorithms

We describe the four machine learning algorithms; KNN, RF, SVM and ANN to be used for classification of *Bactrocera* spp based on morphometrics data.

### K-nearest neighbor

KNN is one of the simplest non-parametric distance-based machine learning algorithms for classification. KNN algorithm assumes the similarity between the new case/data and available cases and put the new case into the category that is most similar to the available categories^18^. KNN selects the number k of the neighbors and calculates a distance measure, commonly Euclidian distance and then assigns the unknown observation to a class based on class majority of the k closest neighbors^11,19^. Thus, k plays an important role in the performance of kNN algorithm and is a key tuning parameter of the model. Herein, the parameter k was determined through cross validation technique, in which different values of k were subjected to the kNN algorithm and the selected k corresponded to the value with the highest accuracy of the model.

### Random forest

Random Forest is a tree-based machine learning technique that leverages the power of multiple decision trees considered as forest in an assemble paradigm for making predictions^20^. A decision tree is a tree-structured classifier, where internal nodes represent the features of a dataset, branches represent the decision rules, and each leaf node represents the outcome. A decision tree has essentially two nodes; decision node and leaf node^20,21^. Decision nodes are used to make decision and have multiple branches, whereas leaf nodes are the output of those decisions and do not contain any further branches. The decisions are performed based on features of the given dataset. The best feature for the root node and for sub-nodes is determined using attribute selection measure**.** A decision tree simply asks a question and based on the answer (Yes/No), it further splits the tree into subtrees. Random forest, as the name suggests, is a “forest” of randomly created decision trees. Each node in the decision tree works on a random subset of features/input variables to calculate the output. The random forest then combines the output of individual decision trees to generate the final output. To implement the random forest, there are two tuning parameters, the number of trees (*ntree*) and the number of features, the input variables in each split (*mtry*). To find the optimal RF model, a range of values for *mtry* parameter were tested and evaluated using repeated cross-validation and the optimal value was selected for which the model accuracy was highest, *ntree* was held constant as 2000.

### Support vector machine algorithm

The goal of Support Vector Machine (SVM) algorithm is to establish the best line or decision boundary that can segregate n-dimensional space into classes that can easily put new subjected data points in the correct category in the future. This best decision boundary is called a hyperplane. SVM chooses the extreme points/vectors that help in creating the hyperplane^22,23^. These extreme cases are referred to as support vectors, and hence the algorithm is termed as support vector machine. There are different kernel functions used in SVM and selecting an appropriate kernel function is crucial for the performance of the SVM. We evaluated the SVM with the simplest kernel, the linear kernel SVM, and two non-linear kernels; the polynomial kernel and the radial basis kernel^24^. Non-linear kernel functions are necessary where samples cannot be separated linearly. There are two parameters that need to be tuned when implementing SVM classifier, thus the optimum parameters of cost, C and the kernel width parameter, gamma (γ). The C parameter decides the size of misclassification allowed for non-separable training data, which makes the adjustment of the rigidity of training data possible. The gamma (**γ**) affects the smoothing of the shape of the class-dividing hyperplane. In this study, C was evaluated using a range of values from 0.01 to 10.0 with step size of 0.25 while γ had values from 0.01 to 0.1 with step size of 0.01. The linear kernel SVM has only one parameter. Optimal values were chosen corresponding to model with highest accuracy.

### Artificial neural network

**Figure 5:**
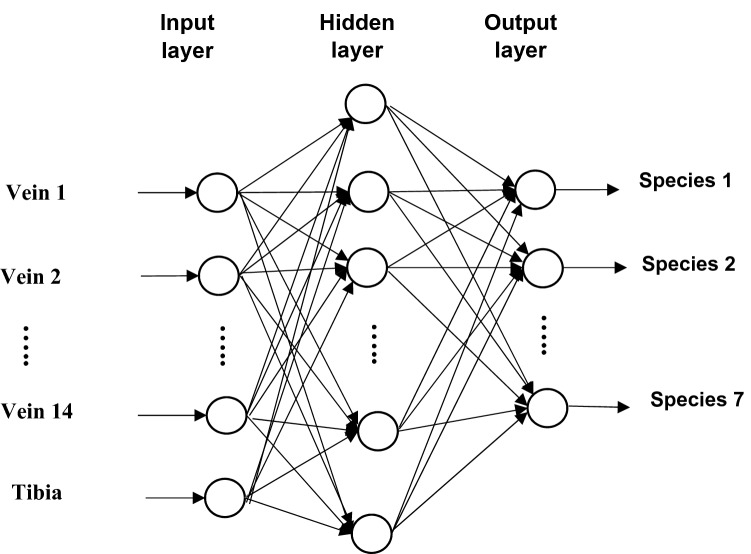
A schematic diagram illustrating the structure of a simple multilayer neural network. Arrows represent the direction that values are passed. At the end of the network, the output layer provides the probability that the specimen in question belongs to a given species.

Artificial neural networks, as the name implies, are inspired from their biological counterparts, the biological brain, and the nervous system. In artificial intelligence, an ANN is based on a collection of connected units or nodes called artificial neurons, which loosely model the neurons in a biological brain^25^. ANN can be applied in supervised and unsupervised training. We use ANN as supervised learning algorithm which means that we provide the input data containing the independent variables and the output data that contains the dependent variable^26,27^. A feed-forward neural network with three layers: input layer, hidden layer and output layer is used (Fig. 5). The back-propagation algorithm, the mostly used optimization technique for the training of feed forward neural networks is used^28^. During data processing, predictions are made in ANN based on the values in the input nodes and the weights, one weight for each input feature. The nodes in the input layer are connected with the output layer via the weight parameters. In the output layer, the values in the input nodes are multiplied with their corresponding weights and are added together. A bias term is added to the sum to improve the level of robustness of the neural network. The sum is passed through an activation function, usually sigmoid activation function:

1 $$f(x) = \frac{1}{{1 + e^{ - \left( x \right)} }}$$

The result of the activation function, Eq. (1) is basically the predicted output for the input features. The back-propagation optimization technique provides the means to adjust the free parameters of the network to minimize error between actual and predicted outcome. In this study, the input layer consists of 15 neurons, the wing vein and tibia length variables and the output layer has 7 neurons, the fruit fly species. The number of neurons for the hidden layer was determined by trial and error.

### Analytics

The data comprise of 1091 observations on 15 morphometric measurements. The output variable are the seven fruit fly species namely; *B*. *correcta* (Bcor), *B. cucurbitae* (Bcu), *B. dorsalis* (Bdo), *B. invadens* (BI), *B. kandiensis* (Bka), *B. oleae* (Bol) and *B. zonata* (Bzo) (Table 5).

The classification algorithms K-Nearest Neighbor, Random Forest, Support Vector Machine (SVM), and Artificial Neural Network (ANN) were trained on 70% of the fruit fly morphometric dataset while 30% of the data was used as test set.

Each model’s performance was evaluated based on accuracy score, Kappa, AUC of the receiver operating characteristics and ‘no- information rate’ (NIR) derived using confusion matrix. A confusion matrix is a table defining the predictive performance of a classifier on a set of test data for which the true values are known. The accuracy is the proportion of samples accurately classified. Kappa statistic reveals how well the model’s predictions match the actual labels of classes while controlling for accuracy of a random classifier. Landis and Koch^29^ classified Kappa statistics within the range of 0.00 and 0.20 as implying poor agreement between classifier’s predictions and the actual labels of the classes; 0.21–0.40 imply fair strength of agreement; 0.41–0.60 imply moderate agreement; 0.61- 0.80 imply substantial strength of agreement while 0.81–1.00 imply an almost perfect agreement. NIR is the score realized by classifier model in predicting the classes when the information beyond the overall distribution of the classes being predicted is unknown. A model with higher NIR than accuracy implies poor performance^30^.

Other model diagnostic metrics on individual outcome classes include sensitivity and specificity. Sensitivity is the rate at which true positives are correctly classified while specificity is the rate at which true negatives are correctly classified.

All statistical analyses were conducted using the R software version 4.0.4^31^. The classification models were implemented using the *caret* package^32^. In addition, the SVM classifier required *kernlab* package^33^ and *e1071* package^34^ while ANN classifier required *neuralnet* package^35^ and *nnet* package^36^. The *ggplot2* package^37^ was used for graphical visualisations. The models were constructed using fivefold cross validation with the hold out fold used to measure the accuracy of each model.

## Supplementary Information

Supplementary Information.

## Data Availability

R code used in this paper is available at http://dmmg.icipe.org/dataportal/dataset/african-fruit-fly-program. The data are available at https://github.com/icipe-official/Machine-Learning-Algorithms-on-Insect-Morphometrics-Data.
