# Supplementary material for: Leveraging machine learning tools and algorithms for analysis of fruit fly morphometrics
Source: Sci Rep. 2022 May 3;12:7208. doi: 10.1038/s41598-022-11258-w (PMC9065030; doi:10.1038/s41598-022-11258-w)
Supplement: Supplementary file 1 — Supplementary Information. [file 41598_2022_11258_MOESM1_ESM.pdf]

Leveraging machine learning tools and algorithms for analysis of fruit fly morphometrics

Daisy Salifu; Eric Ali Ibrahim; Henri E Z Tonnang

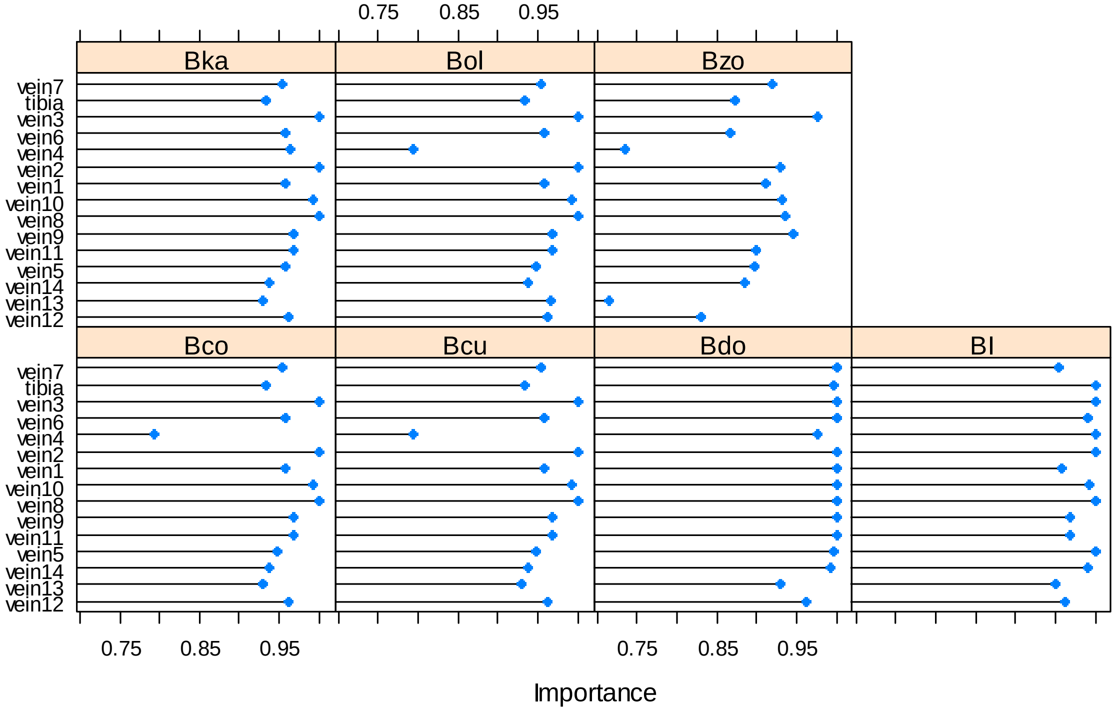

Supplementary Fig. S1. Analysis of variable importance (VI) for the linear kernel SVM model. Veins 3, 2, 8, and 10 are identified as predictors of higher importance than others in all species except for Bdo (*B. dorsalis*) and BI (*B. invadens*).

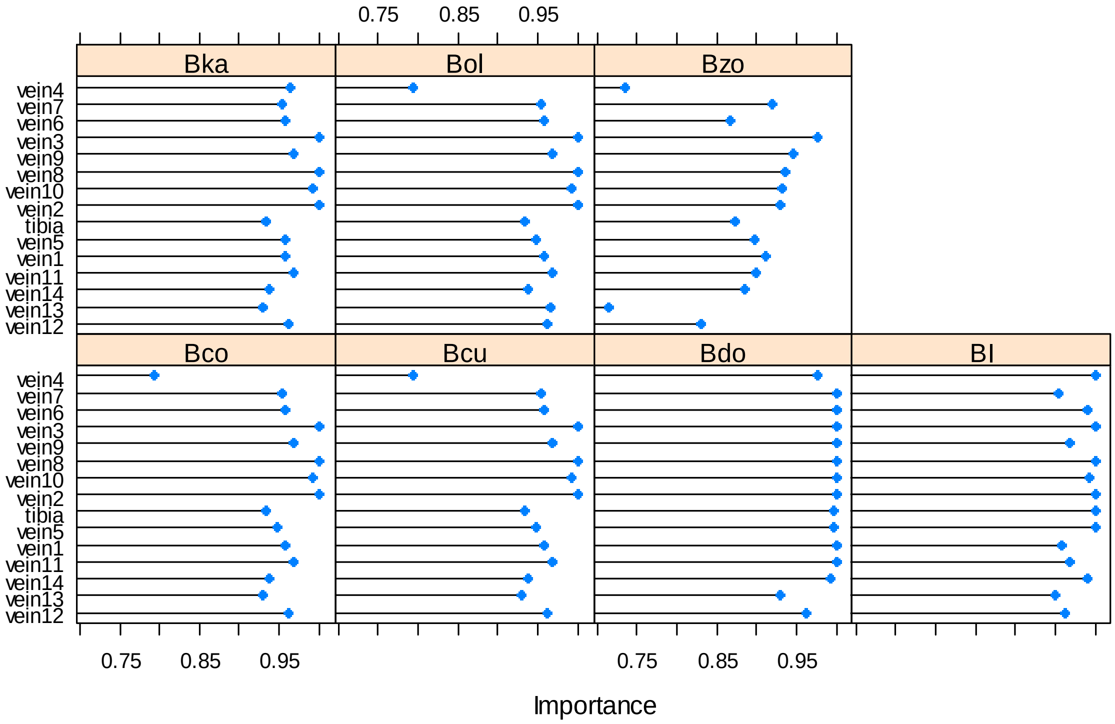

Supplementary Fig. S2. Analysis of variable importance (VI) for the polynomial kernel SVM model. Veins 3, 2, 8, and 10 are identified as predictors of higher importance than others in all species except for Bdo (*B. dorsalis*) and BI (*B. invadens*).
